# Supplementary material for: Toxic or Not Toxic, That Is the Carbon Quantum Dot’s Question: A Comprehensive Evaluation with Zebrafish Embryo, Eleutheroembryo, and Adult Models
Source: Polymers (Basel). 2021 May 15;13(10):1598. doi: 10.3390/polym13101598 (PMC8155906; doi:10.3390/polym13101598)
Supplement: Supplementary file 1 [file polymers-13-01598-s001.zip › polymers-1186167-SI.pdf]

Supporting Information

Toxic or Not Toxic, that is the Carbon Quantum Dot's Question: A Comprehensive Evaluation with Zebrafish Embryo, Eleutheroembryo, and Adult Models

Chih-Yu Chung [1+], Yu-Ju Chen [1+], Chia-Hui Kang [1], Hung-Yun Lin [1], Chih-Ching Huang [1,3], Pang-Hung Hsu [1,2,3]* and Han-Jia Lin [1,3,*]

[1] Department of Bioscience and Biotechnology, National Taiwan Ocean University, Keelung, 20224, Taiwan; jerrych0214@gmail.com (C.-Y. C.); m9541043@gmail.com (Y.-J. C.); kang083035@gmail.com (J.-H. K.); hungyun59@gmail.com (H.-Y. L.); hanjia@ntou.edu.tw (H.-J. L.)

[2] Bachelor Degree Program in Marine Biotechnology, National Taiwan Ocean University, Keelung, 20224, Taiwan; phsu@mail.ntou.edu.tw (P.-H. H.)

[3] Center of Excellence for the Oceans, National Taiwan Ocean University, Keelung, 20224, Taiwan; huanging@ntou.edu.tw (C.-C. H.)

* Correspondence: hanjia@ntou.edu.tw (H.-J. L.), phsu@mail.ntou.edu.tw (P.-H. H.)

+ these authors contributed equally to this work

Table of contents:

Table S1. Epi-characteristics of CQD_{AC} and CQD_{Spd}

Figure S1. The end-point survival rate of FET and FEET

Figure S2. Bright field and fluorescence images of CQDs-soaked embryos after return to normal conditions

Figure S3. Effects of feeding adult zebrafish with CQDs on zebrafish offspring

Table S1. Epi-characteristics of CQD_{AC} and CQD_{Spd}

	Size (nm)^{a*}	Zeta potential (mV)^{b*}	Quantum yield (%)	Diffraction planes	Ref.
**CQD_{AC}**	4.1 ± 1.20	- 41.3 ± 1.70	18.1	graphite (002)	[1,2]
**CQD_{Spd}**	6.3 ± 1.35	+ 45.4 ± 2.80	3.85	graphite (002)	[2,3]

^{a} Average particle sizes are count from TEM images every 100 particles in each CQDs.
^{b} Zeta potential of CQDs in sodium phosphate buffer (pH 7.4, 5 mM).
* Standard deviation of three repeated measurements.

CQD_{AC} is a carbon nanomaterial prepared from ammonium citrate through a pyrolysis reaction, and has a negative surface charge and an average particle size of 4.1 ± 1.20 nm [1,2]. CQD_{Spd} is another carbon nanomaterial made from the polyamine, spermidine, with an average particle size of 6.33 ± 1.35 nm, and a high density of positive charges on its surface [2,3].

Reference

1. Yang Z, Xu M, Liu Y, He F, Gao F, Su Y, Wei H, Zhang Y. Nitrogen-doped, carbon-rich, highly photoluminescent carbon dots from ammonium citrate. Nanoscale. 2014; 6(3): 1890-1895

2. Li YJ, Harroun SG, Su YC, Huang CF, Unnikrishnan B, Lin HJ, et al. Synthesis of self-assembled spermidine-carbon quantum dots effective against multidrug-resistant bacteria. Adv Healthc Mater. 2016; 5(19): 2545-2554

3. Jian HJ, Wu RS, Lin TY, Li YJ, Lin HJ, Harroun SG, Lai JY, Huang CC. Super-cationic carbon quantum dots synthesized from spermidine as an eye drop formulation for topical treatment of bacterial keratitis. ACS Nano. 2017; 11(7): 6703-6716

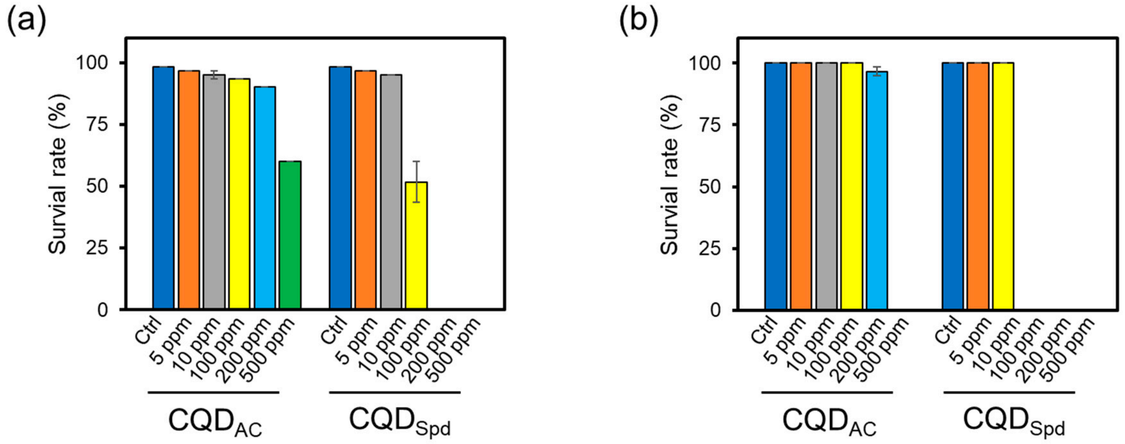

Figure S1. The end-point survival rate of FET and FEET

(a) The survival rates of 0.5-hpf embryo exposed to different concentrations of CQD_{AC} or CQD_{Spd} solution after 96 h. (b) The survival rates of 96-hpf eleutheroembryo exposed to different concentrations of CQD_{AC} or CQD_{Spd} solution after 72 h.

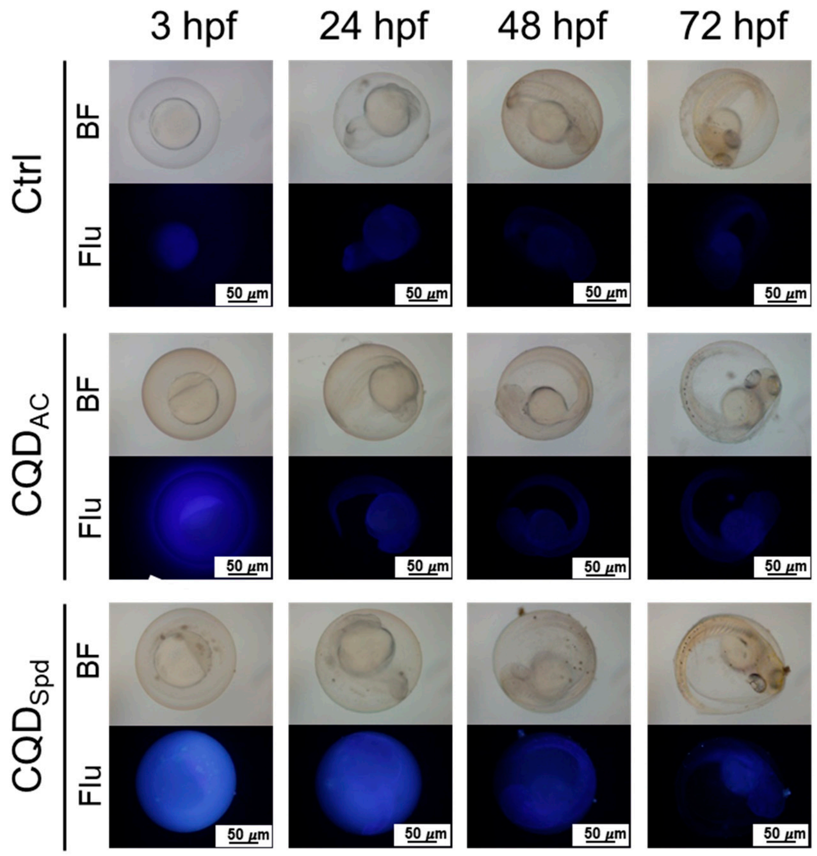

Figure S2. Bright field and fluorescence images of CQDs-soaked embryos after return to normal conditions

The 0.5 hpf embryos were first exposed to 100 ppm CQDs solutions for 3 h. The test medium was subsequently replaced, and microscopic images were taken at 3, 24, 48, and 72 hpf for the observation of the residual fluorescence of CQDs.

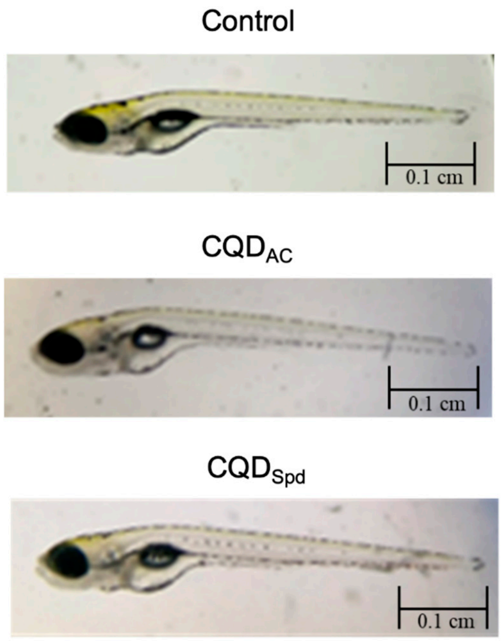

Figure S3. Effects of feeding adult zebrafish with CQDs on zebrafish offspring

The appearances and body lengths of F_1 offspring showed no differences between the control group and CQDs fodders-fed groups; no malformations were observed after one month of rearing F_1 zebrafish offspring.
